# Physiology and Animal Mechanism

**Published:** 1841-11

**Authors:** 


Art. II.—*Physiology and Animal Mechanism.* Philadelphia: 1841: pp. 101.

We have received from the publishers, Turner & Fisher, Philadelphia, a small volume entitled "Physiology and Animal Mechanism," designed for the use of schools and colleges. It is a translation from the French of Milne Edwards and Achille Compte, by Dr. Ruschenberger of the U. S. Navy. We have looked over this little work, and cannot but regard it as preferable to several which have been put forth on the subject of which it treats. From the number of these elementary books, which have been published within the last twelve or fifteen years, it might be supposed that the demand for them has been very great; but such we fear is not the fact. Each has been, to a certain extent, a failure, and its successor has been offered as a work entitled to greater patronage. That none of them have been liberally patronized, is not, perhaps, owing to their defects, so much as the difficulty of introducing a new study into our academies; especially when it presents no *prima facie* claims to utility, in the vulgar acceptation of that term.

We think the medical profession deeply interested in the popular study of physiology; and that they ought, in every way that is practicable, to promote a taste in the people, for this branch of science. Nothing would so effectually repress the encouragement of quackery, and inspire confidence in the medical profession, as a knowledge of physiology.

To this end we recommend Dr. Ruschenberger's little work, in which all the great functions of digestion, respiration, circulation, sensation and locomotion will be found familiarly explained. It is illustrated with plates; but they are on too small a scale, and withal are executed in a rude and imperfect style. This was a great mistake in the publishers; who ought to have known, that in this, as in the study of zoology and geography, more depends on the graphical delineations than on the text. D.

